# Future athletic career direction as a determinant of perceived university conditions for sport

**DOI:** 10.3389/fspor.2026.1786150

**Published:** 2026-05-29

**Authors:** Michal Varmus, Nikola Staffenova, Roman Adamik, Adam Jacko

**Affiliations:** Faculty of Management Sciences and Informatics, Department of Managerial Theories, University of Žilina, Žilina, Slovakia

**Keywords:** dual career, sport conditions, sports management, university studies, youth athletes

## Abstract

**Background:**

The decision of young athletes to study at university is closely related to their future sports direction. Expectations regarding sports conditions at universities can significantly influence their academic and sports decision-making.

**Objective:**

The purpose of the study was to determine to what extent the planned career direction affects the importance that young athletes attribute to sports conditions at university.

**Methods:**

The primary research was conducted through a quantitative study using an online survey, with 396 complete responses included in the analytical processing. The analysis combined non-parametric and parametric procedures: *X*^2^; test with Cramer's V and one-way ANOVA.

**Results:**

The results showed that there is a relationship between sports ambitions, study decisions and assessment of conditions. The greater the role of sports in the future, the greater the emphasis young athletes place on the quality of sports facilities at universities.

**Conclusion:**

This study highlights the importance of sports conditions as an important factor influencing the decision-making process of young athletes when choosing a university. The originality of the article lies in extending the perspective to the pre-university phase and in the fact that expectations regarding sports conditions significantly influence both academic and sports decision-making.

## 1 Introduction

Achieving and maintaining elite-level performance requires long-term investments by athletes and their environment, whether in the areas of physical training, social support or finances (1). The term career does not only mean professional activity in sport, but also a broader sequence of socially significant roles that an individual plays throughout life and that shape their identity, personal fulfillment and professional development (2, 3). A sports career can be understood as a multidimensional developmental process that reflects the interplay between sport and education across different stages of an athlete's life (4). The European Federation of Sport Psychology (5) defines it as multi-year sports activities aimed at high sports performance and self-improvement.

Torregrossa et al. (6) distinguish between linear careers (only sports), convergent (priority sports, simultaneous studies/work), parallel (balance between sports and studies/work) and sports path (performance pressure leads to the termination of one activity). The essential factors influencing the career path are the level of education, the type of socialization and the availability of mentoring (7).

Combining sports and studies is a challenging but key opportunity for career development. It is referred to as a dual career and its success depends on the awareness of its importance by athletes and their surrounding environment (8) and brings benefits such as preventing the closure of sports identity and preparing for life after the end of sports career (9–11). Building a career outside of sport reduces the risk of problems in the labor market (12) and supports employability, coping with career transitions and the development of competencies necessary for effective dual career management (13–15).

Previous research has focused mainly on the transition from junior to senior sport and the support of dual careers during university studies, examining the combination of training and study and the support mechanisms of universities (e.g., 16–20). Less attention has been paid to the period before entering university and how the quality of the sports background influences the choice of school and the planning of a future sports career, as well as the assessment of these conditions by students regarding an active or other career in sport.

The aim of the research is therefore to examine how the preferred future in a sports career influences the ambition to study at university and the importance of conditions for sport that allow for the effective combination of academic obligations and sports activity. The novelty lies in examining the relationship between the educational environment, sports motivation, and career planning in the transition from secondary school to university studies as a key phase in the formation of a dual career.

**Figure 1:**
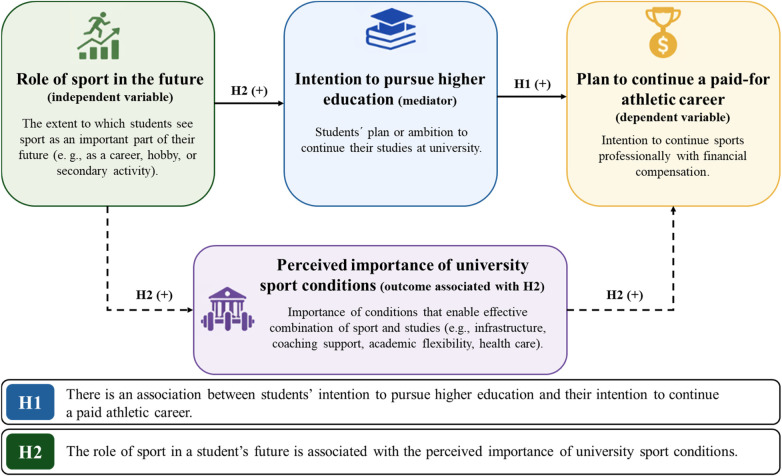
Conceptual model of the relationship between variables and expected directions.

Based on the above, a conceptual model was formulated, which assumes that the preferred direction of a future sports career influences both the ambition to study at a university and the perceived importance of the conditions for sports at the university. These conditions are understood as key factors enabling an effective combination of academic obligations and sports activities. At the same time, it is assumed that a higher orientation towards a sports career is associated with higher demands on the quality and availability of these conditions. The proposed model and the assumed relationships between the variables are schematically illustrated in Figure 1, thus creating a logical framework for the formulation of hypotheses H1 and H2.

In this study, the term “perceived university conditions for sport” is defined as a multifactorial construction that includes the availability and quality of sports infrastructure, access to training and institutional support for sports, flexibility of study that allows for the combination of academic and sports obligations, or access to health and medical care. In simple terms, there are various conditions that are perceived by a given student and are important to him without suggesting specific options. It assumes that each athlete, under appropriate conditions for the type of sport he performs, can imagine something different and at the same time this does not say anything about the real conditions at the university—it is a subjective assumption of the conditions perceived by the student. These factors represent key aspects of the university environment that are related to the development of a dual career of student-athletes.

## 2 Literature review

### 2.1 Junior-to-senior transition and dual career

The transition from youth sports to senior elite athlete represents the most challenging developmental phase of a sports career. Only 20%–30% of young athletes successfully manage it, with many quitting or switching to recreational sports during this period (21–23).

This challenging period involves increased demands on training, performance and psychological resilience (9, 24, 25). Transition can lead to a variety of outcomes, from establishing oneself in senior sport to returning to a recreational level (23, 26–28). Young athletes struggle to balance sport, education and personal life, which increases the risk of stress. Success depends mainly on personal resources such as self-control, self-confidence and sports identity, which influence the efforts invested in a career (28). A strong sports identity supports adaptation at the senior level but can complicate the end of a career (29), while successfully managing the transition is related to the athlete's identity adjustment and personal maturation (9, 10, 30).

Previous research has examined the transition of athletes in different environments and levels: riders from club to regional level (10), individual sports athletes (31), team sports players during the transition from academy to first team (32), hockey players to the elite level (9), or athletes from individual and team sectors throughout the entire career transition period (30, 33).

Education appears to be a key element in building a dual career, especially as the transition from junior to senior age usually corresponds with the completion of high school (17, 34). A dual career implies a simultaneous focus on sport and study or work, while balancing training, peak performance, and preparation for a post-sport career (22, 35, 36).The transition from high school to university for talented athletes is accompanied by higher parental expectations, more intensive training, and biological, psychological, and psychosocial changes associated with puberty (37–40). The development of personal competencies and skills that can achieve excellence in both areas plays an important role in athletes' ability to manage career and life transitions (41–43). Talented and elite athletes who continue to higher secondary and university education struggle to achieve their potential in two areas of success simultaneously (43–45). Ryba et al. (46) investigate the identification of processes and support mechanisms that help athletes successfully navigate the transition to university, a period when their social environment, academic demands, and often sporting goals change. Athletes' motivational profiles are not homogeneous. Aunola et al. (47) identified three stable patterns: dual-career orientation, high sport and low academic orientation, and low sport orientation, with these patterns predicting athletes' future career ambitions.

Continuing to college and obtaining higher education is considered beneficial, especially by athletes who are not willing or able to become professional athletes and make a living from sports (48). The possibility of obtaining a college degree, such as in American college sports, is considered particularly attractive by some athletes (49). Brown et al. (50) and Tekavc et al. (51) also examined the transition from high school to college in athletes, with university education often seen as a “plan B” in case of an end to their sports career. Franck and Stambulova (52) confirm the postponement of college studies due to performance orientation, while Ryba et al. (17) analyze the transition from the perspective of dual careers and international mobility.

Previous research has focused mainly on the transition from junior to senior sport and dual careers, examining how athletes combine training and study. While existing studies examine whether athletes prioritize work or study when transitioning to senior, they do not analyze to what extent they plan to continue in an active paid sports career.

The research gap therefore lies in the lack of knowledge about whether high school athletes plan to continue in college in professional or semi-professional sports, or whether they perceive sports only as a hobby or a path to another career in the sports sector. This question is directly related to hypothesis **H1**: There is an association between students' intention to pursue higher education and their intention to continue a paid athletic career.

### 2.2 Conditions for a sport at university

At some point in their careers, athletes face inevitable changes. One of these is whether to go to university and combine top-level sport with education (10, 17, 18, 20). Universities have a crucial role in creating the conditions that connect sport and academia (19). In the context of this study, these conditions are not treated as separate measurable components, but as a general framework influencing athletes' perceptions of their sporting environment. An effective dual career depends on cooperation between universities, clubs and partners, and problems can hinder the creation of synergies and long-term benefits. A favorable environment and suitable conditions contribute to the highest possible performance of all subjects. Another advantage of studying at a prestigious university is the networking opportunities beyond sport, which help build social capital for athletes (15).

Athletes' experiences may vary depending on the demands of the sport environment and the culture in which they operate (9, 53). Personalized programs at universities can respond to the specific educational needs that these students have (16). Mateu et al. (54) report that the more important sport is, the more students value an environment that supports the maintenance of a sports identity during their studies.

Athletes consider the high workload of combining sports and studies to be a problem, especially when there are insufficient university support and a preference for flexible schedules over optimal preparation for a future career (38, 55, 56).

An athlete can continue an active career if adequate dual career policies are implemented at the university (57); however, it is reported that approximately 60%–70% of student-athletes lack sufficient institutional support for dual careers, with greater reliance placed on support from parents and friends. The importance of both positive and negative influences from family, teammates, friends and institutions is also highlighted in previous research (10, 58, 98).

The following aspects are discussed as part of the broader conceptual background of perceived university sport conditions. Academic and athletic success depends on multiple factors. In addition to academic support, adequate training conditions, medical, health and social care (59), digitalization of processes (60), organizational leadership and psychological support (15) and infrastructure that allows the alignment of training and academic schedules (61, 62) are also important. The cooperation of the university with the sports club is also important (38, 50). Negative biases from educators and other students can complicate dual careers, so understanding teachers and existing assistance programs that help athletes balance academic and athletic commitments are key (63–67).

Previous research has focused mainly on supporting dual careers during university and on programs that allow for combining training and education. Less attention has been paid to how sporting conditions influence school choices even before entering university. There is a lack of knowledge about how students evaluate these conditions in relation to their future sporting ambitions, whether professional, semi-professional, coaching or otherwise.

In this study, these aspects are reflected in a single evaluative judgment of the importance of university sport conditions, rather than being assessed as separate constructs. The identified research gap emphasizes the need to investigate to what extent athletes perceive the importance of university sports background depending on their planned career path, with the importance of conditions increasing for those for whom sport has a significant role in the future. This is the focus of hypothesis **H2:** The role of sport in a student's future is associated with the perceived importance of university sport conditions.

## 3 Methodology

### 3.1 Data collection

The primary research was conducted through a quantitative study using our own online survey. Quantitative survey studies have been used by several authors on the topic of dual careers and athlete education. López de Subijana et al. (68) analysed the obstacles faced by Spanish elite athletes in combining a sports career and studies. Chamorro et al. (69) focused on the motivation and goals of youth elite soccer players during the transition from junior to senior sport. Healy et al. (70) examined the relationships between university athletes' sports and academic goals and their intrinsic motivation.

The questionnaire was developed specifically for this study, drawing on established frameworks of dual career research and sports career typology as described by Stambulova et al. (22) and Torregrossa et al. (6). Items were formulated to address three core dimensions identified in the literature: career orientation, educational planning, and the perceived importance of the university sport environment. All items were reviewed by the research team for content adequacy prior to distribution. A pilot test conducted with twelve student-athletes confirmed the clarity and comprehensibility of the questions.

The questionnaire included both background variables and attitudinal items; however, the key construction of perceived university sport conditions was measured separately using a single-item scale. The questionnaire consisted of 25 questions focused on the basic characteristics of the respondents and their attitudes towards sports and studies. Some of the questions were answered using a 5-point Likert scale, others were closed questions with predefined answers, and some were open-ended. The sampling approach can be characterized as a non-probability convenience sampling method based on voluntary participation through sports clubs and sports schools.

It investigated demographic data (age, gender, place of residence, type of high school, sports branch) and the role of sports in the future lives of the respondents, i.e., whether they plan a professional or semi-professional career (active paid-for form), amateur activity or another sports career (e.g., coach, referee, manager). The respondents also indicated whether they plan to continue at university and how important they attach to the conditions for sports that the university provides.

Data collection was carried out via Google Forms and was distributed to youth sports clubs or sports clubs with a youth component and sports high schools in Slovakia in February to June 2025. Secondary sports schools and sports clubs in Slovakia were approached, where teachers and coaches were responsible for distributing the questionnaire. The final data set included 396 valid responses. According to the calculation, the minimum sample size for the base set (*n* = 55,000) is 384 responses. A more detailed sample calculation is provided in Chapter 3.2.

### 3.2 Sample

The research was conducted on a sample of young athletes from Slovak sports clubs active in both individual and team sports aged 14 to 19, which is a key period for making decisions about a professional sports career. The size of the base population (*n* = 55,000) was based on data on the number of active athletes in each age group (71) and trends in the number of students enrolled in lower secondary education (72). The representative sample with data for analytical evaluation (*n* = 396) shows a deviation of 4.91%.

In terms of gender, 31.06% of respondents were female (*n* = 123), 68.43% male (*n* = 271) and 0.51% did not indicate gender (*n* = 2). In terms of age, 30.56% of participants were older than 18 years (*n* = 121) and 69.44% were younger than 18 years (*n* = 275).

Among team sports, football dominated (37.12%, *n* = 147), followed by handball (11.11%, *n* = 44) and hockey (9.34%, *n* = 37). Among individual sports, tennis (3.79%, *n* = 15), swimming and water sports (3.79%, *n* = 15) and athletics (2.27%, *n* = 9) were most frequently represented.

In terms of the type of secondary school, most respondents attended grammar schools (38.13%, *n* = 151), secondary vocational schools (37.12%, *n* = 147) and secondary sports schools (23.74%, *n* = 94). The sample reflects an even and balanced distribution of respondents across gender, sport types, and educational backgrounds relevant to youth sport participation.

### 3.3 Hypotheses

The article formulates two hypotheses aimed at better understanding the factors influencing students’ decision to pursue a sports career after high school. Existing studies focus mainly on the support of dual careers during university studies, while the decision-making period before entering university and the factors influencing the continuation of active sports are only marginally investigated (10, 54, 55). The hypotheses therefore reflect the need to understand how academic ambitions and future career expectations shape these decisions.

Previous research shows that university environments can offer athletes organizational, psychological, and social support that significantly facilitates continuation in sports (15, 59). Students entering university may also provide an opportunity to pursue a professional or semi-professional sports career, especially if suitable dual career programs are available (63, 65). This suggests that the intention to pursue higher education is associated with a higher likelihood of pursuing a paid-for sports career.

**H1:** There is an association between students' intention to pursue higher education and their intention to continue a paid athletic career.

Research also confirms the importance and significance of the university environment in terms of opportunities to combine sports and studies, especially in the areas of infrastructure, training conditions, staff support and organizational flexibility (61, 62). These conditions are particularly important for athletes who expect sport to play a significant role in their future identity or career (54). Therefore, it is hypothesized that the importance of university conditions depends on the planned intensity of sports involvement.

**H2:** The role of sport in a student's future is associated with the perceived importance of university sport conditions.

### 3.4 Data analysis

The analytical part of the research was designed to capture the connections between the respondents' sports ambitions, their educational decisions, and the environment of preparation for a sports future. The variables required a combination of statistical approaches typical of sports-educational research, with an emphasis on sensitivity to categorized and ordinal data. The analytical procedure drawn from Aliberti et al. (73) was structured to identify categorical relationships between variables, detect directional differences between groups, and assess the practical magnitude of observed effects. As grounded in sports-behavioral research by Byshevets et al. (74) and Field (75), this approach enables examination of the complex decision-making patterns of youth athletes regarding their practical significance, as further demonstrated by Silva et al. (76).

#### 3.4.1 Measurement properties and reliability considerations

The career ambition items used a four-point ordinal response scale. Standard Cronbach's alpha assumes interval-level data and systematically underestimates reliability for ordinal variables. Ordinal alpha based on polychoric correlations was therefore computed as the appropriate reliability estimate, following Zumbo et al. (77). Polychoric correlations between the three career ambition items ranged from .258 to .683, with all pairwise associations statistically significant, *p* < .001.

Ordinal alpha for the three career ambition items was α_ord = .696. For the two paid career items specifically, the value reached α_ord = .812, reflecting their stronger conceptual overlap. The somewhat lower three-item value is consistent with the nature of the items. Professional and semi-professional career orientations are conceptually closer to each other than either is to non-playing sports roles. The other sports career item was therefore analysed as a separate outcome rather than as part of a composite scale.

To examine the construct structure, an exploratory factor analysis using polychoric correlations was conducted. A single-factor solution was supported by the Kaiser criterion. Factor 1 yielded an eigenvalue of 1.895 and accounted for 63.2% of common variance. No further factors met the retention threshold. Factor loadings were .856 for professional career ambition, .894 for semi-professional career ambition, and .603 for other sports career ambition. The pattern confirms a shared underlying dimension of sports career orientation across all three items. The perceived importance of university sport conditions was captured through a single evaluative item. As Diamantopoulos et al. (78) demonstrate, internal consistency reliability is not applicable to single-item measures. For this item, construct validity rests on its theoretical grounding rather than on internal consistency, and this limitation is acknowledged in Section 5.4. The item draws on dual career research identifying the university sport environment as a key determinant of athlete decision-making and career continuation, as demonstrated by Mateu et al. (54) and contextualised within broader career transition frameworks by Stambulova et al. (22).

#### 3.4.2 Chi-square test of independence and Cramér's V

Following the considerations regarding measurement properties outlined in Section 3.4.1, it was necessary to determine whether there were non-random relationships between the categories. The chi-square test of independence was used to analyze the categorized variables, which can capture differences in the distribution of responses between groups without assuming normality of the data (75). Its suitability is also confirmed by Singh and Purohit (79) in examining physical activity and Song et al. (80) in assessing sports habits. To interpret the intensity of the relationships, it was supplemented by the Cramér's V indicator, which considers the number of categories and sample size and allows assessing the practical significance of the differences (74, 81).

#### 3.4.3 One-way ANOVA

The chi-square test identifies the existence of relationships between variables but does not provide information about the direction of differences between groups. For this reason, one-way analysis of variance was used to compare differences in ambition levels between groups. Although ANOVA is traditionally intended for interval data, it is commonly used in sports research to analyze ordinal variables and Likert scales with sufficiently large sample sizes, where it demonstrates robustness to moderate violations of normality (74, 82). The interpretation of results was supplemented with effect size indicators.

Prior to each analysis of variance, homogeneity of variances was examined using Levene's test. In the H1 analyses, variances were homogeneous across all groups. For the professional career variable, the test yielded W = 1.233, *p* = .293, and for the semi-professional career variable W = 0.223, *p* = .801, confirming the appropriateness of standard ANOVA in both cases. In the H2 analyses, Levene's test indicated heterogeneous variances, with *p* = .006 for the professional career variable and *p* < .001 for the semi-professional and other sports career variables. Given the unequal group sizes in the H2 data, Kruskal–Wallis tests were conducted as a non-parametric robustness check for all three H2 subanalyses. In all cases, the direction and significance of results were consistent between ANOVA and Kruskal–Wallis, supporting the reliability of the parametric findings despite the variance heterogeneity.

The combination of the chi-square test and ANOVA thus allows identification not only of whether relationships exist between variables, but also of their direction and the magnitude of group differences. Where analysis of variance indicated statistically significant group differences, pairwise *post-hoc* comparisons were conducted using Tukey's HSD test to identify which specific groups drove the observed effects.

#### 3.4.4 Effect size indicators (*η*², *ω*², Cramér's V)

In addition to testing statistical significance, effect size indicators were also used to assess the extent of the differences and relationships found between variables. Effect size measures provide information about the practical significance of the results, which is particularly important in the social and sports sciences, where statistically significant but practically weak effects can occur in large sample sizes.

Eta squared (*η*²) and omega squared (*ω*²) were used to express effect size in analysis of variance (ANOVA) and indicate the proportion of the variability in the dependent variable explained by the corresponding group variable. Cramér's V was used to measure the strength of the association between categorical variables in the chi-square test.

The use of effect size measures allows for a more comprehensive interpretation of the results by supplementing statistical significance with information about the strength and practical relevance of the observed relationships.

## 4 Results

This section presents the results of analyses examining the relationship between sports career ambitions, university plans, and the perceived importance of university sport conditions. For the H1 analyses, respondents were grouped according to their stated university intention into three categories: planned to attend university, undecided, and did not plan to attend university. For the H2 analyses, respondents were grouped according to their rating of the importance of university sport conditions on a six-point scale ranging from cannot say to very important. Results are reported separately for each hypothesis. For each analysis, the chi-square test was applied first to establish whether a statistically significant association existed, followed by one-way ANOVA to examine the direction and magnitude of group differences.

### 4.1 The relationship between paid-for career and ambition for higher education (H1)

The analysis examined the relationship between the intention to pursue a paid sports career and the plan to study at university. The aim was to examine the relationship between academic decision-making and the motivation to continue in professional or semi-professional sport. First, the statistical association of the variables was tested, then the level of sports ambitions was compared according to the plan of university studies. The analysis was carried out separately for professional and semi-professional ambitions to reveal differences according to the career level.

#### 4.1.1 Professional career and university studies

The chi-square test confirmed a statistically significant association between professional career ambitions and university plans, *χ*² (6, *N* = 396) = 13.480, *p* = .036, with Cramér's V of .130 indicating a weak relationship. Descriptive and inferential statistics are presented in Table 1.

**Table 1:** Descriptive and inferential statistics for H1: career ambitions by university plans.

Descriptive statistics					Inferential statistics						
Career type	Group	*n*	M	SD	*χ*² (df)	*p*	V	F (df)	p	*η*²	*ω*²
Professional career	Planned to attend	271	2.653	1.16	13.480 (6)	.036	.130	5.853 (2, 393)	.003	.029	.024
	Undecided	86	3.116	1.056							
	Did not plan	39	2.949	1.169							
Semi-professional career	Planned to attend	271	2.59	0.91	30.747 (6)	<.001	.197	2.883 (2, 393)	.057	.014	.009
	Undecided	86	2.744	0.857							
	Did not plan	39	2.333	0.838							

*N* = 396; M, mean; SD, standard deviation; V, Cramér's V; *η*^2^, eta squared; ω², omega squared. Career ambition: 4-point scale (1 = do not wish; 4 = most desired). Groups: Planned to attend = intend to attend university; Undecided = do not yet know; Did not pla*n* = do not intend to attend. Levene's test: professional W = 1.233, *p* = .293; semi-professional W = 0.223, *p* = .801 — homogeneous variances. *post-hoc* comparisons (professional) in Table 3. Light grey = significant ANOVA. Dark grey = non-significant. Effect sizes: *η*^2^ ≥ .01 small, ≥ .06 medium (99).

One-way ANOVA revealed significant differences in ambition scores across the three groups, F (2, 393) = 5.853, *p* = .003, *η*² = .029, *ω*² = .024. The undecided group reported the highest ambition score, M = 3.116, SD = 1.056, followed by those who did not plan to attend university, M = 2.949, SD = 1.169, and students planning to attend university, M = 2.653, SD = 1.160.

Tukey's HSD *post-hoc* test identified one significant pairwise difference between the undecided and planned-to-attend groups, *p* = .003, with no other pairs reaching significance. The direction of the hypothesis was not confirmed. Students planning to attend university showed the lowest ambition scores rather than the highest. The undecided group stood out as the most ambitious, which suggests that unresolved academic plans may be associated with broader and less constrained career expectations. Complete pairwise comparisons are presented in Table 2.

**Table 2:** Tukey HSD *post-hoc* pairwise comparisons for H1—professional career ambitions.

Group A	n(A)	M(A)	SD(A)	Group B	n(B)	M(B)	SD(B)	*p* (Tukey)	Significant?
Planned to attend	271	2.653	1.16	Undecided	86	3.116	1.056	.003	Yes^*^
Planned to attend	271	2.653	1.16	Did not plan	39	2.949	1.169	.285	No
Undecided	86	3.116	1.056	Did not plan	39	2.949	1.169	.727	No

*F* (2, 393) = 5.853, *p* = .003. Only undecided vs. planned to attend reached significance. Light grey = *p* < .05.

“*” means that statistical significance = *p* < .05.

#### 4.1.2 Semi-professional career and university studies

The chi-square test confirmed a statistically significant association between semi-professional career ambitions and university plans, *χ*^2^ (6, *N* = 396) = 30.747, *p* < .001, with Cramér's V of .197 indicating a weak relationship. Despite the significant chi-square result, one-way ANOVA did not reveal statistically significant differences in ambition scores across the three groups, F (2, 393) = 2.883, *p* = .057, *η*² = .014, *ω*² = .009. Descriptive and inferential statistics are presented in Table 1.

Mean scores were similar across all three groups. The undecided group scored highest, M = 2.744, SD = 0.857, followed by students planning to attend university, M = 2.590, SD = 0.910, and those who did not plan to attend, M = 2.333, SD = 0.838. Given that ANOVA did not reach significance, *post-hoc* comparisons were not conducted. Semi-professional ambitions appear relatively evenly distributed regardless of university plans. The chi-square result reflects a non-random pattern in the categorical distribution of responses, but this pattern does not translate into meaningful differences in ambition levels between groups.

#### 4.1.3 Overall evaluation of the results for H1

Both analyses confirmed a statistically significant association between career ambitions and university plans, though the relationships were weak in terms of effect size. For professional career ambitions, ANOVA identified significant group differences driven primarily by the undecided group, which reported higher ambitions than students planning to attend university. For semi-professional ambitions, the chi-square result was significant, but ANOVA did not confirm meaningful differences between groups.

Hypothesis H1 was not confirmed in its directional form. The data do not support the assumption that students planning to attend university show higher paid career ambitions. The decision to pursue higher education appears to be shaped by factors beyond athletic ambition alone, and the undecided group in particular warrants closer attention in future research.

### 4.2 The relationship between the future role of sport and the importance of conditions for sport at university (H2)

The second part of the analysis examined the relationship between the planned role of sport in the future and the importance of sports facilities at university. The aim was to determine whether students with higher ambitions who plan to continue in an active career place greater emphasis on the quality of training and support facilities at university. First, the statistical association between the variables was verified, then the level of sports ambitions was compared according to the assessment of the importance of university facilities. The analysis was carried out separately for professional, semi-professional and alternative careers to reveal differences between the groups. It should be noted that a substantial proportion of respondents selected the cannot say response category, which may reflect genuine uncertainty about the role of university conditions rather than indifference, and this distributional characteristic should be considered when interpreting the findings.

#### 4.2.1 Professional career and the importance of conditions for sports at university

The chi-square test confirmed a statistically significant association between professional career ambitions and the perceived importance of university sport conditions, *χ*^2^ (15, *N* = 396) = 42.787, *p* < .001, with Cramér's V of .190 indicating a weak to moderate relationship. One-way ANOVA revealed significant differences in ambition scores across the six conditions groups, F (5, 390) = 5.296, *p* < .001, *η*^2^ = .064, *ω*^2^ = .051. The Kruskal–Wallis test yielded consistent results, H = 26.568, *p* < .001, *ε*^2^ = .055, confirming the robustness of the parametric findings. Descriptive and inferential statistics are presented in Table 3.

**Table 3:** Descriptive and inferential statistics for H2: career ambitions by perceived importance of university sport conditions.

Descriptive statistics					Inferential statistics								
Career type	Conditions group	*n*	M	SD	*χ*² (df)	*p*	V	F (df)	*p*	*η*²	*ω*²	KW H	KW p/*ε*²
Professional career	Cannot say	212	2.736	1.163	42.787 (15)	<.001	.190	5.296 (5, 390)	<.001	.064	.051	26.568	<.001/.055
	Not important	9	1.889	0.928									
	Neutral	42	2.452	1.152									
	Somewhat important	46	3.391	1.043									
	Important	61	2.689	1.041									
	Very important	26	3.154	1.12									
Semi-professional career	Cannot say	212	2.519	0.961	53.025 (15)	<.001	.211	1.407 (5, 390)	.221	.018	.005	8.111	.150/.008
	Not important	9	2.333	1									
	Neutral	42	2.548	0.968									
	Somewhat important	46	2.761	0.639									
	Important	61	2.738	0.681									
	Very important	26	2.808	0.981									
Other sports career	Cannot say	212	2.255	1.017	69.753 (15)	<.001	.242	3.045 (5, 390)	.010	.038	.025	14.234	.014/.024
	Not important	9	1.556	0.882									
	Neutral	42	2.524	1.087									
	Somewhat important	46	1.978	0.614									
	Important	61	2.295	0.901									
	Very important	26	2.615	1.098									

*N* = 396; M, mean; SD, standard deviation; V, Cramér's V; *η*², eta squared; *ω*², omega squared; KW H, Kruskal–Wallis statistic; *ε*², epsilon squared. Conditions scale: 1 = Cannot say; 2 = Not important; 3 = Neutral; 4 = Somewhat important; 5 = Important; 6 = Very important. KW tests conducted as robustness checks (Levene: professional *p* = .006; semi-professional and other sports *p* < .001). ANOVA and KW consistent in all subanalyses. Post-hoc: professional in Table 4, other sports career in Table 5. Light grey = significant ANOVA. Dark grey = non-significant. *η*² ≥ .01 small, ≥.06 medium (99).

The highest ambition scores were reported by respondents who rated university conditions as somewhat important, M = 3.391, SD = 1.043, and very important, M = 3.154, SD = 1.120. The lowest score was found in the group that considered conditions not important, M = 1.889, SD = 0.928.

Tukey's HSD *post-hoc* test identified five significant pairwise differences. The somewhat important group scored significantly higher than they cannot say group, *p* = .005, the not important group, *p* = .004, and the neutral group, *p* = .002. The somewhat important group also scored significantly higher than the important group, *p* = .018. Additionally, the not important group scored significantly lower than the very important group, *p* = .044. Complete pairwise comparisons are presented in Table 4.

**Table 4:** Tukey HSD *post-hoc* pairwise comparisons for H2—professional career ambitions.

Group A	M (A)	SD (A)	Group B	M (B)	SD (B)	Diff (A−B)	q	*p* (Tukey)	Significant?
Cannot say	2.736	1.163	Not important	1.889	0.928	+0.847	3.134	.233	No
Cannot say	2.736	1.163	Neutral	2.452	1.152	+0.283	2.113	.668	No
Cannot say	2.736	1.163	Somewhat important	3.391	1.043	−0.655	5.074	.005	Yes^*^
Cannot say	2.736	1.163	Important	2.689	1.041	+0.047	0.41	.999	No
Cannot say	2.736	1.163	Very important	3.154	1.12	−0.418	2.533	.473	No
Not important	1.889	0.928	Neutral	2.452	1.152	−0.563	1.932	.747	No
Not important	1.889	0.928	Somewhat important	3.391	1.043	−1.502	5.19	.004	Yes^*^
Not important	1.889	0.928	Important	2.689	1.041	−0.800	2.82	.348	No
Not important	1.889	0.928	Very important	3.154	1.12	−1.265	4.119	.044	Yes^*^
Neutral	2.452	1.152	Somewhat important	3.391	1.043	−0.939	5.54	.002	Yes^*^
Neutral	2.452	1.152	Important	2.689	1.041	−0.236	1.483	.901	No
Neutral	2.452	1.152	Very important	3.154	1.12	−0.701	3.54	.126	No
Somewhat important	3.391	1.043	Important	2.689	1.041	+0.703	4.532	.018	Yes^*^
Somewhat important	3.391	1.043	Very important	3.154	1.12	+0.237	1.219	.955	No
Important	2.689	1.041	Very important	3.154	1.12	−0.465	2.502	.487	No

*F* (5, 390) = 5.296, *p* < .001. Five significant pairs, all involving the Somewhat important group. Light grey = *p* < .05.

“*” means that statistical significance = *p* < .05.

Students with stronger professional career ambitions consistently placed greater importance on the quality of university sport conditions, which aligns with the theoretical expectation that athletes oriented toward a paid career are more attuned to the institutional environment supporting their development.

#### 4.2.2 Semi-professional career and the importance of conditions for sports at university

The chi-square test confirmed a statistically significant association between semi-professional career ambitions and the perceived importance of university sport conditions, *χ*^2^ (15, *N* = 396) = 53.025, *p* < .001, with Cramér's V of.211. One-way ANOVA did not reveal statistically significant differences in ambition scores across the six groups, F (5, 390) = 1.407, *p* = .221, *η*^2^ = .018, *ω*^2^ = .005. The Kruskal–Wallis test yielded a consistent non-significant result, H = 8.111, *p* = .150, *ε*^2^ = .008. Descriptive and inferential statistics are presented in Table 3.

Mean ambition scores ranged narrowly across all groups. The very important group scored highest, M = 2.808, SD = 0.981, followed by the somewhat important group, M = 2.761, SD = 0.639, while the not important group reported the lowest score, M = 2.333, SD = 1.000. Given the non-significant ANOVA result, *post-hoc* comparisons were not conducted.

The chi-square result reflects a non-random pattern in the categorical distribution of responses, but this pattern does not translate into meaningful differences in ambition levels. Semi-professional ambitions appear relatively uniform across respondents regardless of how they rated university sport conditions.

#### 4.2.3 Different careers in sports and the importance of conditions for sports at university

The chi-square test confirmed a statistically significant association between other sports career ambitions and the perceived importance of university sport conditions, *χ*^2^ (15, *N* = 396) = 69.753, *p* < .001, with Cramér's V of.242 indicating a moderate relationship. One-way ANOVA revealed statistically significant differences in ambition scores across the six groups, F (5, 390) = 3.045, *p* = .010, *η*^2^ = .038, *ω*^2^ = .025. The Kruskal–Wallis test yielded a consistent result, H = 14.234, *p* = .014, *ε*^2^ = .024. Descriptive and inferential statistics are presented in Table 3.

The highest ambition score was reported by the very important group, M = 2.615, SD = 1.098, while the lowest was found among respondents who considered conditions not important, M = 1.556, SD = 0.882. The neutral group, M = 2.524, SD = 1.087, and they cannot say group, M = 2.255, SD = 1.017, occupied intermediate positions. Although ANOVA indicated a significant overall effect, Tukey's HSD *post-hoc* test did not identify any individually significant pairwise differences. This pattern is consistent with a diffuse effect in which no single pair of groups accounts for the overall variance, a finding that is itself informative in suggesting that orientation toward non-playing sport roles is broadly rather than specifically associated with sensitivity to university sport conditions. Complete pairwise comparisons are presented in Table 5.

**Table 5:** Tukey HSD *post-hoc* pairwise comparisons for H2—other sports career ambitions.

Group A	M (A)	SD (A)	Group B	M (B)	SD (B)	Diff (A−B)	q	*p* (Tukey)	Significant?
Cannot say	2.255	1.017	Not important	1.556	0.882	+0.699	2.988	.283	No
Cannot say	2.255	1.017	Neutral	2.524	1.087	−0.269	2.317	.574	No
Cannot say	2.255	1.017	Somewhat important	1.978	0.614	+0.276	2.472	.501	No
Cannot say	2.255	1.017	Important	2.295	0.901	−0.040	0.404	.999	No
Cannot say	2.255	1.017	Very important	2.615	1.098	−0.361	2.524	.477	No
Not important	1.556	0.882	Neutral	2.524	1.087	−0.968	3.834	.075	No
Not important	1.556	0.882	Somewhat important	1.978	0.614	−0.423	1.687	.840	No
Not important	1.556	0.882	Important	2.295	0.901	−0.740	3.012	.274	No
Not important	1.556	0.882	Very important	2.615	1.098	−1.060	3.985	.057	No
Neutral	2.524	1.087	Somewhat important	1.978	0.614	+0.546	3.718	.093	No
Neutral	2.524	1.087	Important	2.295	0.901	+0.229	1.659	.850	No
Neutral	2.524	1.087	Very important	2.615	1.098	−0.092	0.534	.999	No
Somewhat important	1.978	0.614	Important	2.295	0.901	−0.317	2.36	.554	No
Somewhat important	1.978	0.614	Very important	2.615	1.098	−0.637	3.777	.084	No
Important	2.295	0.901	Very important	2.615	1.098	−0.320	1.989	.723	No

*F* (5, 390) = 3.045, *p* = .010. Overall ANOVA significant but no pairwise comparison reached significance. Closest: Not important vs. Very important, *p* = .057. Diffuse effect across multiple small differences.

Respondents oriented toward non-playing roles in sport, such as coaching, officiating or sport management, appear sensitive to the quality of the university sport environment. They likely perceive institutional conditions as relevant not only for athletic performance but also for professional preparation in their intended career path.

#### 4.2.4 Overall evaluation of results for H2

Across all three career types, chi-square tests confirmed statistically significant associations between career ambitions and the perceived importance of university sport conditions. The strength of these associations varied, with Cramér's V ranging from .190 for professional ambitions to .242 for other sports career ambitions. ANOVA results were consistent with the Kruskal–Wallis robustness checks in all three subanalyses.

Directional differences were confirmed for professional and other sports career ambitions, while semi-professional ambitions showed a significant categorical association without meaningful group-level differences in ANOVA. The pattern across all three analyses suggests that the more prominent the role of sport in a respondent's future, the greater the sensitivity to the quality of the university sport environment.

Hypothesis H2 was confirmed. The role of sport in a student's future is associated with the perceived importance of university sport conditions, though the nature and strength of this relationship differ across career types.

## 5 Discussion

### 5.1 Summary of main results

The results of the study provide an expanded perspective on the decision-making of young athletes when transitioning to college and show that the plan to continue their studies alone does not explain the level of sporting ambition as clearly as the original hypothesis assumed. The highest ambitions were shown by athletes who had not yet chosen whether to go to college, which shows that the transition period is very diverse and that athletes' decisions depend on a combination of their personal abilities and external circumstances (10, 28, 30). This finding is also consistent with Aunola et al. (47), who showed that athletes with high sport, but low academic orientation display the highest sport ambitions, which may explain why undecided respondents in this study reported the highest levels of ambition. Subjective experiences and emotional experiences of the transition to a higher level of performance can lead to differences in career paths even among athletes with similar conditions (9, 53).

The hypothesis about the relationship between the importance of sport in the future and the emphasis on the quality of sports conditions was confirmed. Students with higher ambitions paid more attention to infrastructure, individual services and flexible learning conditions, which is in line with the findings of Mateu et al. (54). The research shows that the importance of sporting conditions is crucial even before university choice, complementing the findings of Guidotti et al. (83) on the lack of research on pre-university determinants of decision-making.

The discrepancy between the expected direction of the hypothesis and the results suggests that assumptions about the relationship between academic continuation and sporting ambitions are overly simplistic. Similarly, the literature on emotional and contextual factors of transitions shows that athletes may respond to periods of uncertainty with increased motivation or decreased engagement (9).

Overall, the study shows that decision-making about the future before entering university is a dynamic process that cannot be explained by a single variable. This can be further interpreted through the uncertainty of career transitions, which are not linear but involve multiple possible trajectories and outcomes. Transition periods in sport are therefore characterized by instability and decision-making dilemmas, where athletes may simultaneously maintain high sporting ambitions but do not have a clear future educational or career path (23, 26, 27, 28). This uncertainty is further influenced by the structural characteristics of education systems, where the emphasis on sports performance, the uneven availability of academic support, and the varying quality of educational programs may influence the extent to which athletes prioritize or postpone educational decisions (84, 85). Furthermore, the transition from junior to senior sport is associated with increased performance demands, training load and psychological pressure, especially in team sports, where athletes must also adapt to new group dynamics and expectations in the club environment (9, 25, 100). Economic and resource conditions also play a significant role, as elite sport places high financial and time demands on families, including transportation, equipment and long-term support, which influences both educational and sporting decisions (86–90). This pattern suggests that decision-making about future educational and sporting pathways is influenced not only by individual perceptions of the transition, but also by broader contextual conditions. At the same time, it confirms that quality sports conditions are a key motivational factor for athletes with the ambition to continue an active career and expands existing dual career models with knowledge about pre-university determinants.

### 5.2 Academic implications

The findings show that the pre-university phase is indeed crucial and significantly influences the ambitions and decision-making of young athletes, thus extending the theoretical frameworks of Ryba et al. (17). The transition between junior and senior levels is much more diverse than traditional models assume (9, 30), and the highest sporting ambitions paradoxically appear in athletes who do not yet know whether they will go to university. This suggests that sporting identity, academic plans and expectations for the future may not always be in line.

The importance of university sporting conditions is already evident in the school selection phase, not only during the dual career, as previously assumed (91–93). Furthermore, it appears that what is crucial is not the specific planning of a professional or semi-professional career (57), but the overall role of sport in the respondent's future life.

These findings complement existing dual career models with new decision-making mechanisms and suggest that current theoretical approaches need to be updated to better reflect the complex and less linear reality of sports trajectories.

### 5.3 Practical implications

The practical recommendations arising from the research are relevant to universities, sports clubs and policy makers. Universities should clearly communicate their sports conditions, training opportunities, infrastructure and support programs to high school students, inspired by the NCAA approach (94–96), as these factors influence school choice. Attention should be paid to undecided students who show the highest sporting ambitions and may need targeted academic and sports support to maintain a career in sport and develop a dual career.

Integrated guidance and flexible learning conditions, which include organisational, psychosocial and infrastructural aspects (15, 61, 62, 97), are essential to enable athletes to effectively balance training and studies. Collaboration between universities and sports clubs should start at the pre-university stage, which helps young athletes to choose the right study programme and supports the development of a long-term sports career (38, 50).

These recommendations highlight the need for a systematic approach that considers academic ambitions, sporting goals and a suitable environment before entering university.

### 5.4 Limitations and future research

The research has several limitations that are necessary when interpreting the results. The first limitation is the fact that the research was based on self-reported responses of respondents, which can be influenced by subjective perception and socially desirable responses (self-report bias). Another limitation is the one-time data collection (cross-sectional design), which allows for the analysis of only current attitudes and declared intentions, not their development over time. For this reason, the respondents' threats and decisions may change in the future, while longitudinal research should provide more accurate information about their development.

Also, the limitation is the use of a convenience sample (within a single country), which may reduce the possibility of generalizing the results to other countries or different sports and education systems (single-country bias). In addition, the uneven representation of individual sports and school types may partially influence the results.

Another methodological limitation is the use of a single measurement of a key construct (single-item scale), which limits the possibility of verifying its reliability and the broader dimensions of the construct. Although it was chosen with the aim of an overall focused assessment, future research could have used multi-item scales to measure individual dimensions in more detail.

Since the research was conducted in an environment of minor respondents, ethical aspects of data collection are also needed, including informed consent and potential inaccuracy of answers influenced by understanding the questions or social pressure. Another limitation is the absence of qualitative data that could have further explained why some “non-athletes” achieve the highest standards.

Future research should therefore expand the design to include longitudinal follow-up of respondents, use multi-item and validated scales, include more countries to increase the generalizability of results, add the perspective of parents, coaches and teachers who influence the decision-making of young athletes.

## 6 Conclusion

The research has provided new insights into how young athletes consider combining sports and academic careers before entering university and fills a gap in the existing literature in this decision-making period. It has been shown that although there is a connection between planned studies and sports ambitions, this relationship does not manifest itself in the way predicted by dual career theory. The highest ambitions were among undecided athletes, which underlines the complexity of decision-making processes in the transition period between high school and university.

At the same time, it has been confirmed that the importance of university sports conditions increases with the increased importance of sport in the future. This finding extends the existing literature to a new phase of decision-making, namely the period before entering university, which has been investigated to a lesser extent in previous research.

The limitations of the research point to the need for longer-term follow-ups, sample expansion and the involvement of other relevant actors. Future research should focus on a deeper understanding of athletes' motivations and the creation of models that better capture the variability of sports and academic decisions.

The article thus makes an important contribution to the knowledge of young athletes' decision-making, identifies new determinants of university choice, and provides recommendations for the creation of more effective policies and support programs for dual careers.

## Data Availability

The original contributions presented in the study are included in the article/Supplementary Material, further inquiries can be directed to the corresponding author.

## References

[1] Wylleman P. The career development of elite athletes: a sport psychological perspective. During the 4th International Scientific Conference on Kinesiology: Science and Profession—challenge for the Future. Opatija, Croatia: University of Zagreb (2005). p. 622–7. ISBN 953-6378-52-3

[2] Betz NE. Career self-efficacy: exemplary recent research and emerging directions. J Career Assess. (2007) 15(4):403–22. 10.1177/1069072707305759

[3] Brusokas A Malinauskas R. Career self-efficacy among lithuanian adolescents in sports schools. Proc Soc Behav Sci. (2014) 116:212–6. 10.1016/j.sbspro.2014.01.196

[4] Quinaud RT Doupona M Guidotti F Capranica L. Editorial: multidimensional development of student-athletes: new perspectives on dual career. Front Sports Act Living. (2025) 7:1701681. 10.3389/fspor.2025.1701681 41048630 PMC12491260

[5] FEPSAC—European Federation of Sport Psychology. Sports career transitions. (1997). Available online at: https://fepsac.com/position-statements/ (Accessed November 21, 2025).

[6] Torregrossa M Chamorro JL Prato L Ramis Y. Grupos, entornos y carrera deportiva. In: Blanch TI Humanidades T, editors. Dirección de equipos deportivos. Valencia: Tirant lo Blanc (2021). p. 255–371. ISBN 978-84-18614-36-1.

[7] M’mbaha JM Chepyator-Thomson JR. Factors influencing career paths and progress of Kenyan women in sport leadership. Qual Res Sport Exerc Health. (2018) 11(3):316–33. 10.1080/2159676X.2018.1446042

[8] Vidal-Vilaplana A Staskeviciute-Butiene I Pérez-Campos C González-Serrano MH. Student-athletes and sports stakeholders’ perceptions on the EdMedia educational platform for training and promoting dual career on social Media. Cult Cienc Deporte. (2025) 20(63):2237. 10.12800/ccd.v20i63.2237

[9] Bruner MW Munroe-Chandler KJ Spink KS. Entry into elite sport: a preliminary investigation into the transition experiences of rookie athletes. J Appl Sport Psychol. (2008) 20(2):236–52. 10.1080/10413200701867745

[10] Pummell B Harwood C Lavallee D. Jumping to the next level: a qualitative examination of within-career transition in adolescent event riders. Psychol Sport Exerc. (2008) 9(4):427–47. 10.1016/j.psychsport.2007.07.004

[11] Stambulova N Franck A Weibull F. Assessment of the transition from junior-to-senior sports in Swedish athletes. Int J Sport Exerc Psychol. (2012) 10(2):79–95. 10.1080/1612197X.2012.645136

[12] Stambulova N Harwood C. A “Dual Career”: combining sport and studies. Front Young Minds. (2022) 10:692422. 10.3389/frym.2022.692422

[13] De Brandt K Wylleman P Torregrossa M Schipper-Van Veldhoven N Minelli D Defruyt S. Exploring the factor structure of the dual career competency questionnaire for athletes in European pupil- and student-athletes. Int J Sport Exerc Psychol. (2018) 23(3):1–18. 10.1080/1612197X.2018.1511619

[14] Linnér L Stambulova N Lindahl K Wylleman P. Swedish University student-athletes’ dual career scenarios and competences. Int J Sport Exerc Psychol. (2019) 23(3):37–52. 10.1080/1612197X.2019.1611898

[15] Belluzzi M Ferraboli A Gozzoli C D’Angelo C. The experience of Italian student athletes enrolled in a dual career university program: the challenges of employability. Front Sports Act Living. (2025) 6:1515634. 10.3389/fspor.2024.1515634 39850869 PMC11754183

[16] Álvarez Pérez PR Pérez-Jorge D López Aguilar D González Herrera AI. Transición y adaptación a los estudios universitarios de los deportistas de alto nivel: la compleja relación entre aprendizaje y práctica deportiva. REOP Revista Española de Orientación y Psicopedagogía. (2014) 25(2):74–89. 10.5944/reop.vol.25.num.2.2014.13521

[17] Ryba TV Stambulova N Ronkainen N Bundgaard J Selänne H. Dual career pathways of transnational athletes. Psychol Sport Exerc. (2015) 21:125–34. 10.1016/j.psychsport.2014.06.002

[18] Defruyt S Wylleman P Kegelaers J De Brandt K. Factors influencing Flemish elite athletes’ decision to initiate a dual career path at higher education. Sport Soc. (2020) 23(4):660–77. 10.1080/17430437.2019.1669324

[19] Vidal-Vilaplana A Valantine I Staskeviciute-Butiene I González-Serrano MH Capranica L Calabuig F. Combining sport and academic career: exploring the current state of student-athletes’ dual career research field. J Hosp, Leis, Sport & Tour Educ. (2022) 31:1–14. 10.1016/J.JHLSTE.2022.100399

[20] Grafnetterova N Ortega G Del Real Viramontes J. Experiences of 2-year transfer athletes: “There should be More of an Education Process.”. Community Coll J Res Pract. (2023) 49(3):169–86. 10.1080/10668926.2023.2283600

[21] De Martelaer K Rzewnicki R De Knop P Wylleman P Vanden Auweele Y. (Ed.) Parents and coaches: a help or harm? Affective outcomes for children in sport. In: Vanden Auweele Y, editors. Ethics in Youth Sport: Analyses and Recommendations. Leuven: LannooCampus (2004). p. 223. ISBN 90-209-5919-0

[22] Stambulova N Alfermann D Statler T Côté J. ISSP Position stand: career development and transitions of athletes. Int J Sport Exerc Psychol. (2009) 7(4):395–412. 10.1080/1612197X.2009.9671916

[23] Franck A Stambulova N Ivarsson A. Swedish Athletes’ adjustment patterns in the junior-to-senior transition. Int J Sport Exerc Psychol. (2016) 16(4):398–414. 10.1080/1612197X.2016.1256339

[24] Rosier N Wylleman P De Bosscher V Van Hoecke J. The transition from junior to senior athlete: a comparison of successful versus unsuccessful transitions. During the 28th International Congress of Applied Psychology. Paris, France: IAAP (2014). 10.07.

[25] Stambulova NB Pehrson S Olsson K. Phases in the junior-to-senior transition of Swedish ice hockey players: from a conceptual framework to an empirical model. Int J Sports Sci Coach. (2017) 12(2):231–44. 10.1177/1747954117694928

[26] Morris R Tod D Oliver E. An analysis of organizational structure and transition outcomes in the youth-to-senior professional soccer transition. J Appl Sport Psychol. (2015) 27(2):216–34. 10.1080/10413200.2014.980015

[27] Storm LK. “Coloured by culture": talent development in scandinavian elite sport as seen from a cultural perspective (Ph.D. Thesis). Syddansk Universitet. Det Sundhedsvidenskabelige Fakultet, Odense (2015), p. 256.

[28] Franck A Stambulova N Weibull F. Profiles of personal characteristics and relevant pathways in the junior-to-senior transition. A longitudinal study of Swedish athletes. Int J Sport Psychol. (2016) 47(6):483–507. 10.7352/IJSP.2016.47.483

[29] Stephan Y Brewer BW. Perceived determinants of identification with the athlete role among elite competitors. J Appl Sport Psychol. (2007) 19(1):67–79. 10.1080/10413200600944090

[30] Stambulova N. Talent development in sport: a career transitions perspective. In: Tsung-Min Hung E Lidor R & Hackfort D, editors. Psychology of Sport Excellence. Morgantown, WV: Fitness Information Technology (2009). p. 63–74. ISBN 9781885693907.

[31] Pummell E Lavallee D. Preparing UK tennis academy players for the junior-to-senior transition: development, implementation, and evaluation of an intervention program. Psychol Sport Exerc. (2019) 40:156–64. 10.1016/j.psychsport.2018.07.007

[32] Finn J McKenna J. Coping with academy-to-first-team transitions in elite english male team sports: the Coaches’ perspective. Int J Sports Sci Coach. (2010) 5(2):257–79. 10.1260/1747-9541.5.2.257

[33] Stambulova N. Developmental sports career investigations in Russia: a post-perestroika analysis. Sport Psychol. (1994) 8:221–37. 10.1123/tsp.8.3.221

[34] Hickey C Kelly P. Professional Education and Training for Early Career Players in the Australian Football League: Footy First, Second and Third (Version 1). Melbourne: Deakin University (2005). p. 1–13. Available online at: https://hdl.handle.net/10536/DRO/DU:30005929 (Accessed November 21, 2025).

[35] Li M Sum RKW. A meta-synthesis of elite athletes’ experiences in dual career development. Asia Pacific J Sport Soc Sci. (2017) 6(2):99–117. 10.1080/21640599.2017.1317481

[36] Stambulova N Wylleman P. Dual career development and transitions (editorial). In: Stambulova N Wylleman P, editors. Special Issue “Dual Career Development and Transitions”, Psychology of Sport and Exercise. Amsterdam: Elsevier Ltd. (2015), Vol. 21. p. 1–3. 10.1016/j.psychsport.2015.05.003

[37] Newman BM Lohman BJ Myers MC Newman PR. Experience of urban youth navigating the transition to ninth grade. Youth and Society. (2000) 31(–):387–416. 10.1177/0044118X00031004001

[38] Giacobbi PR Jr. Lynn TK Wetherington JM Jenkins J Bodendorf M Langley B. Stress and coping during the transition to university for first-year female athletes. Sport Psychol. (2004) 18(1):1–20. 10.1123/tsp.18.1.1

[39] Wylleman P Lavallee D. A developmental perspective on transitions faced by athletes. In: Weiss MR, editor. Developmental Sport and Exercise Psychology: A Lifespan Perspective. California: Fitness Information Technology (2004). p. 503–23. ISBN 978-1885693365.

[40] Cosh S Tully PJ. “All I have to do is pass”: a discursive analysis of student athletes’ talk about prioritising sport to the detriment of education to overcome stressors encountered in combining elite sport and tertiary education. Psychol Sport Exerc. (2014) 15:180–9. 10.1016/j.psychsport.2013.10.015

[41] Elbe AM Beckmann J. Motivational and self-regulatory factors and sport performance in young elite athletes. In: Hackfort D Tenenbaum G, editors. Essential Processes for Attaining Peak Performance. Michigan: Oxford, Meyer & Meyer (2006). p. 137–56. ISBN 9781841264874.

[42] Baron-Thiene A Alfermann D. Personal characteristics as predictors for dual career dropout versus continuation—a prospective study of adolescent athletes from German elite sport schools. Psychol Sport Exerc. (2015) 21:42–9. 10.1016/j.psychsport.2015.04.006

[43] Stambulova N Engström C Franck A Linnér L Lindahl K. Searching for an optimal balance: dual career experiences of Swedish adolescent athletes. Psychol Sport Exerc. (2015) 21:4–14. 10.1016/j.psychsport.2014.08.009

[44] Lally PS Kerr GA. The career planning, athletic identity, and student role identity of intercollegiate student athletes. Res Q Exerc Sport. (2005) 76(3):275–85. 10.1080/02701367.2005.10599299 16270705

[45] O’Neill M Allen B Calder AM. Pressures to perform: an interview study of Australian high performance school-age athletes’ perceptions of balancing their school and sporting lives. Perform Enhanc Health. (2013) 2:87–93. 10.1016/j.peh.2013.06.001

[46] Ryba TV Aunola K Kalaja S Selänne H Ronkainen NJ Nurmi JE. A new perspective on adolescent athletes’ transition into upper secondary school: a longitudinal mixed methods study protocol. Cogent Psychol. (2016) 3(1):1–15. 10.1080/23311908.2016.1142412

[47] Aunola K Selänne A Selänne H Ryba TV. The role of adolescent athletes’ task value patterns in their educational and athletic career aspirations. Learn Individ Differ. (2018) 63(2018):34–43. 10.1016/j.lindif.2018.03.004

[48] Aquilina D. A study of the relationship between elite Athletes’ educational development and sporting performance. Int J Hist Sport. (2013) 30(4):374–92. 10.1080/09523367.2013.765723

[49] McCormack C Walseth K. Combining elite women’s soccer and education: Norway and the NCAA. Soc Soc. (2013) 14(6):887–97. 10.1080/14660970.2013.843927

[50] Brown DJ Fletcher D Henry I Borrie A Emmett J Buzza A. A British university case study of the transitional experiences of student-athletes. Psychol Sport Exerc. (2015) 21:78–90. 10.1016/j.psychsport.2015.04.002

[51] Tekavc J Wylleman P Cecić Erpič S. Perceptions of dual career development among elite level swimmers and basketball players. Psychol Sport Exerc. (2015) 21:27–41. 10.1016/j.psychsport.2015.03.002

[52] Franck A Stambulova N. The junior to senior transition: a narrative analysis of the pathways of two Swedish athletes. Qual Res Sport Exerc Health. (2018) 11(3):284–98. 10.1080/2159676X.2018.1479979

[53] Morris R Tod D Eubank M. From youth team to first team: an investigation into the transition experiences of young professional athletes in soccer. Int J Sport Exerc Psychol. (2017) 15(5):523–39. 10.1080/1612197X.2016.1152992

[54] Mateu P Inglés E Torregrossa M Marques RFR Stambulova N Vilanova A. Living life through sport: the transition of elite Spanish student-athletes to a university degree in physical activity and sports sciences. Front Psychol. (2020) 11:1367. 10.3389/fpsyg.2020.01367 32655454 PMC7325594

[55] Wylleman P Reints A. A lifespan perspective on the career of talented and elite athletes: perspectives on high-intensity sports. Scand J Med Sci Sport. (2010) 20:88–94. 10.1111/j.1600-0838.2010.01194.x 20840566

[56] Debois N Ledon A Wylleman P. A lifespan perspective on the dual career of elite male athletes. Psychol Sport Exerc. (2015) 21:15–26. 10.1016/j.psychsport.2014.07.011

[57] Silva JVP Quinaud RT Grubertt GA Costa FR Figueiredo AJ. Specific processes for admission of athletes to undergraduate programs at Brazilian federal universities. Front Educ. (2025) 10:1585694. 10.3389/feduc.2025.1585694

[58] Anderson ML Goodman J Schlossberg NK. Counseling Adults in Transition: Linking Schlossberg’s Theory with Practice in a Diverse World. 4th ed. New York: Springer Publishing Company (2012). p. 360. ISBN 978-0826106353

[59] Costa FR Figueiredo AJ. Reflexões sobre a dupla carreira—a harmonia entre a universidade pública e o esporte de alto rendimento. Revista da ALESDE. (2021) 13(1):1–16. 10.5380/jlasss.v13i1.79904

[60] Koman G Toman D Jankal R Boršoš P. The importance of e-recruitment within a smart government framework. Systems. (2024) 12(3):71. 10.3390/systems12030071

[61] Guirola I Torregrosa M Ramis Y Jaenes JC. Remando contracorriente: facilitadores y barreras para compaginar el deporte y los estudios. Rev Andaluza Med Deporte. (2016) 11(1):12–7. 10.1016/j.ramd.2016.08.002

[62] Pérez-Rivasés A Torregrossa M Pallarès S Viladrich C Regüela S. Seguimiento de la transición a la universidad em mujeres deportistas de alto rendimiento. Rev Psicol Deporte. (2017) 26(3):102–7. ISSN: 1132-239X.

[63] Geraniosova K Ronkainen N. The experience of dual career through Slovak Athletes’ eyes. Phys Cult Sport Stud Res. (2015) 66(1):53–64. 10.1515/pcssr-2015-0005

[64] Mateu P Vilanova A Andrés A Inglés E. Más allá de la carrera deportiva. Satisfacción percibida por estudiantes-deportistas sobre un programa universitario de apoyo a la carrera dual. Rev Esp Educ Fís Deportes. (2018) 421:49–58. 10.55166/reefd.vi421.665

[65] Mateu P Vilanova A Inglés E. Análisis de las características organizativas de los programas de apoyo a estudiantes-deportistas de élite en el sistema universitario de Cataluña. Movimento. (2018) 24(4):1205–18. 10.22456/1982-8918.82235

[66] Vickers E. An examination of the dual career pathway and transitions UK student-athletes experience throughout university education (Doctoral thesis). Liverpool John Moores University, Liverpool, United Kingdom (2018). p. 312. 10.24377/LJMU.t.00008878

[67] Torregrossa M Reguela S Mateos M. Career assistance programs. In: Hackfort D Schinke R, editors. The Routledge International Encyclopaedia of Sport and Exercise Psychology. London: Routledge (2020). p. 1–35. ISBN 9781315187228

[68] López de Subijana C Barriopedro M Conde E. Supporting dual career in Spain: elite athletes’ barriers to study. Psychol Sport Exerc. (2015) 21:57–64. 10.1016/j.psychsport.2015.04.012

[69] Chamorro JL Torregrosa M Sánchez Oliva D García Calvo T León B. Future achievements, passion and motivation in the transition from junior-to-senior sport in Spanish young elite soccer players. Span J Psychol. (2016) 19:E69. 10.1017/sjp.2016.71 27762186

[70] Healy L Ntoumanis N Duda J. Goal motives and multiple-goal striving in sport and academia: a person-centered investigation of goal motives and inter-goal relations. J Sci Med Sport. (2016) 19(12):1010–4. 10.1016/j.jsams.2016.03.001 27025304

[71] Greguška I Blažo M. Počet aktívnych športovcov. Jeden z parametrov vzorca na výpočet príspevku uznanému športu alebo prvok kreativity? (2022). Available online at: www.epi.sk/odborny-clanok/pocet-aktivnych-sportovcov-jeden-z-parametrov-vzorca-na-vypocet-prispevku-uznanemu-sportu-alebo-prvok-kreativity.htm (Accessed November 21, 2025).

[72] Eurostat. Pupils enrolled in lowersecondary education by programme orientation, sex and age. (2025). Available online at: https://ec.europa.eu/eurostat/databrowser/product/page/educ_uoe_enrs02 (Accessed November 21, 2025).

[73] Aliberti S D’Elia F Cherubini D. Tips for statistical tools for research methods in exercise and sport sciences. Phys Educ Theory Methodol. (2023) 23(3):470–7. 10.17309/tmfv.2023.3.20

[74] Byshevets N Shynkaruk O Stepanenko O Gerasymenko S Tkachenko S Synihovets I. Development skills implementation of analysis of variance at sport-pedagogical and biomedical researches. J Phys Educ Sport. (2019) 19(6):2086–90. 10.7752/jpes.2019.s6311

[75] Field A. Discovering Statistics Using IBM SPSS Statistics. 5th ed. London: SAGE Publications (2018). p. 1561. ISBN 978-1526419521

[76] Silva AF Conte D Clemente FM. Decision-making in youth team-sports players: a systematic review. Int J Environ Res Public Health. (2020) 17(11):3803. 10.3390/ijerph17113803 32471126 PMC7312689

[77] Zumbo BD Gadermann AM Zeisser C. Ordinal versions of coefficients alpha and theta for Likert rating scales. J Mod Appl Stat Methods. (2007) 6(1):21–9. 10.22237/jmasm/1177992180

[78] Diamantopoulos A Sarstedt M Fuchs C Wilczynski P Kaiser S. Guidelines for choosing between multi-item and single-item scales for construct measurement: a predictive validity perspective. J Acad Mark Sci. (2012) 40(3):434–49. 10.1007/s11747-011-0300-3

[79] Singh A Purohit B. Evaluation of global physical activity questionnaire (GPAQ) among healthy and obese health professionals in central India. Balt J Health Phys Act. (2011) 3(1):34–43. 10.2478/v10131-011-0004-6

[80] Song G Yan Y Zhao H Chen J Deng Y Zhu W. A questionnaire study on the knowledge, attitudes, and practices of fluid replacement and urination among Chinese elite athletes. PLoS One. (2022) 17(10):e0275685. 10.1371/journal.pone.0275685 36223380 PMC9555643

[81] Kelly A Wilson MR Jackson DT Goldman DE Turnnidge J Côté J. A multidisciplinary investigation into “playing-up” in academy football according to age phase. J Sports Sci. (2020) 39(8):854–64. 10.1080/02640414.2020.1848117 33203302

[82] Eldridge J Palmer TB Gillis K Lloyd R Squires WG Murray TD. Comparison of academic and behavioral performance between athletes and non-athletes. Int J Exerc Sci. (2014) 7(1):3–13. 10.70252/UVXP4768 27182397 PMC4831893

[83] Guidotti F Cortis C Capranica L. Dual career of European student-athletes: a systematic literature review. Kinesiol Slov. (2015) 21(3):5–20. ISSN 1318-2269.

[84] Zhang Y Chin JW Reekie SHM. Education in the Chinese national sport system: experiences of professional wushu athletes. Sport Soc. (2019) 22(8):1466–80. 10.1080/17430437.2018.1529168

[85] Fahrner M Burk V. Relevance of university dual career support services—student–athletes’ perspectives. Manag Sport Leis. (2025) 30(3):436–51. 10.1080/23750472.2023.2191614

[86] Gould D Lauer L Rolo C Jannes C Pennisi N. Understanding the role parents play in tennis success: a national survey of junior tennis coaches. Br J Sports Med. (2006) 40:632–6. 10.1136/bjsm.2005.024927 16702176 PMC2564313

[87] Gould D Lauer L Rolo C Jannes C Pennisi N. The role of parents in tennis success: focus group interviews with junior coaches. Sport Psychol. (2008) 22(1):18–37. 10.1123/tsp.22.1.18

[88] Harwood C Knight C. Understanding parental stressors: an investigation of British tennis-parents. J Sports Sci. (2009) 27(4):339–51. 10.1080/02640410802603871 19191064

[89] Lauer L Gould D Roman N Pierce M. Parental behaviors that affect junior tennis player development. Psychol Sport Exerc. (2010) 11(6):487–96. 10.1016/j.psychsport.2010.06.008

[90] Lauer L Gould D Roman N Pierce M. How parents influence junior tennis players’ development: qualitative narratives. J Clin Sport Psychol. (2010) 4(1):69–92. 10.1123/jcsp.4.1.69

[91] Stambulova N Wylleman P. Athletes’ career development and transitions. In: Papaioannou AG Hackfort D, editors. Routledge Companion to Sport and Exercise Psychology: Global Perspectives and Fundamental Concepts. London: Routledge/Taylor & Francis Group (2014). p. 605–20.

[92] Vickers E Morris R. Pathway decisions during the student-athlete transition out of university in the United Kingdom. J Appl Sport Psychol. (2021) 34(4):803–24. 10.1080/10413200.2021.1884918

[93] Donald WE Baruch Y Ashleigh MJ. Construction and operationalisation of an employability capital growth model (ECGM) via a systematic literature review (2016–2022). Stud High Educ. (2023) 49(1):1–15. 10.1080/03075079.2023.2219270

[94] Flaherty M Sagas M. Early recruiting in NCAA sport: an exploratory study of scarcity effects. J Study Sports Athletes Educ. (2020) 14(3):165–91. 10.1080/19357397.2020.1759354

[95] Soltis SM Sterling C Ferrier WJ Borgatti S. College football recruiting: the role of relational rivalry in factor-market competition. Group Organ Manag. (2024) 51(3):1–35. 10.1177/10596011241273377

[96] Corr C. The thematic emphasis of NCAA official recruiting visits during the COVID-19 pandemic. J Study Sports Athletes Educ. (2024) 19(2):162–74. 10.1080/19357397.2024.2321827

[97] Wylleman P Reints A De Knop P. A developmental and holistic perspective on athletic career development. In: Sotiaradou P De Bosscher V, editors. Managing High Performance Sport. New York, NY: Routledge (2013). p. 159–82. 10.4324/9780203132388

[98] Falls D Wilson B. ‘Reflexive modernity’ and the transition experiences of university athletes. Int Rev Sociol Sport. (2012) 48(5):572–93. 10.1177/1012690212445014

[99] Cohen J. Statistical Power Analysis for the Behavioral Sciences (2nd ed.). New York, New York: Routledge (1988). p. 567. 10.4324/9780203771587

[100] Rosier N Wylleman P De Bosscher V Van Hoecke J. The transition from junior to senior elite athlete. In: Proceedings 14th European Congress of Sport Psychology. Bern (2015). p. 157–157.

